# Countdown to 2015 country case studies: systematic tools to address the “black box” of health systems and policy assessment

**DOI:** 10.1186/s12889-016-3402-5

**Published:** 2016-09-12

**Authors:** Neha S. Singh, Luis Huicho, Hoviyeh Afnan-Holmes, Theopista John, Allisyn C. Moran, Tim Colbourn, Chris Grundy, Zoe Matthews, Blerta Maliqi, Matthews Mathai, Bernadette Daelmans, Jennifer Requejo, Joy E. Lawn, On behalf of the Countdown to 2015 Health Systems and Policies Technical Working Group

**Affiliations:** 1 Centre for Maternal, Adolescent, Reproductive and Child Health, London School of Hygiene & Tropical Medicine, London, WC1E 7HT UK; 2 Centro de Investigación para el Desarrollo Integral y Sostenible, Universidad Peruana Cayetano Heredia, Lima, Peru; 3 School of Medicine, Universidad Nacional Mayor de San Marcos, Lima, Peru; 4 Instituto Nacional de Salud del Niño, Lima, Peru; 5 Independent consultant, London, UK; 6 World Health Organisation, PO Box 9292, Dar es Salaam, Tanzania; 7 US Agency for International Development, Bureau of Global Health, Office of Health, Infectious Disease and Nutrition, Washington DC, USA; 8 Institute for Global Health, University College London, London, SW7 2AZ UK; 9 Division of Social Statistics and Demography, University of Southampton, Highfield, Southampton SO17 1BJ UK; 10 Department of Maternal, Newborn, Child and Adolescent Health, World Health Organisation, Geneva 27, 1211 Switzerland; 11 Partnership for Maternal, Newborn & Child Health, Geneva 27, 1211 Switzerland

**Keywords:** Policy analysis, Health systems, Reproductive health, Newborn health, Maternal health, Child health, Tanzania, Peru

## Abstract

**Background:**

Evaluating health systems and policy (HSP) change and implementation is critical in understanding reproductive, maternal, newborn and child health (RMNCH) progress within and across countries. Whilst data for health outcomes, coverage and equity have advanced in the last decade, comparable analyses of HSP changes are lacking. We present a set of novel tools developed by Countdown to 2015 (Countdown) to systematically analyse and describe HSP change for RMNCH indicators, enabling multi-country comparisons.

**Methods:**

International experts worked with eight country teams to develop HSP tools via mixed methods. These tools assess RMNCH change over time (e.g. 1990–2015) and include: (i) *Policy and Programme Timeline Tool* (depicting change according to level of policy*)*; (ii) *Health Policy Tracer Indicators Dashboard* (showing 11 selected RMNCH policies over time); (iii) *Health Systems Tracer Indicators Dashboard* (showing four selected systems indicators over time); and (iv) Programme implementation assessment. To illustrate these tools, we present results from Tanzania and Peru, two of eight Countdown case studies.

**Results:**

The Policy and Programme Timeline tool shows that Tanzania’s RMNCH environment is complex, with increased funding and programmes for child survival, particularly primary-care implementation. Maternal health was prioritised since mid-1990s, yet with variable programme implementation, mainly targeting facilities. Newborn health only received attention since 2005, yet is rapidly scaling-up interventions at facility- and community-levels. Reproductive health lost momentum, with re-investment since 2010. Contrastingly, Peru moved from standalone to integrated RMNCH programme implementation, combined with multi-sectoral, anti-poverty strategies.

The HSP Tracer Indicators Dashboards show that Peru has adopted nine of 11 policy tracer indicators and Tanzania has adopted seven. Peru costed national RMNCH plans pre-2000, whereas Tanzania developed a national RMNCH plan in 2006 but only costed the reproductive health component. Both countries included all lifesaving RMNCH commodities on their essential medicines lists. Peru has twice the health worker density of Tanzania (15.4 vs. 7.1/10,000 population, respectively), although both are below the 22.8 WHO minimum threshold.

**Conclusions:**

These are the first HSP tools using mixed methods to systematically analyse and describe RMNCH changes within and across countries, important in informing accelerated progress for ending preventable maternal, newborn and child mortality in the post-2015 era.

**Electronic supplementary material:**

The online version of this article (doi:10.1186/s12889-016-3402-5) contains supplementary material, which is available to authorised users.

## Background

The Millennium Development Goals (MDGs) ended in 2015, when the 189 signatory countries assessed progress made in the past 15 years. At the heart of the MDGs are MDG4, which called for a reduction of child mortality by two-thirds, and MDG5, which focused on improvement of maternal health through a reduction of maternal mortality by three-quarters and universal access to reproductive health care [1].

**Fig. 1:**
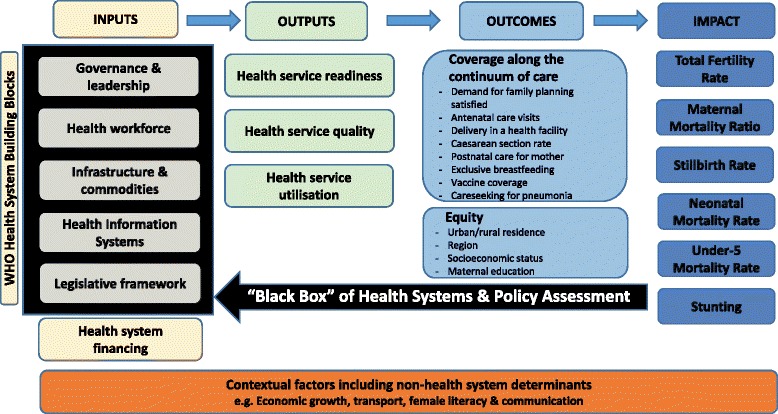
Evaluation framework for Countdown to 2015 country case studies

Established in 2005, Countdown to 2015 for Maternal, Newborn and Child Survival (Countdown) uses country-specific data to stimulate and support country progress towards achieving MDG4 and MDG5 in the 75 countries where more than 95 % of all maternal, newborn and child deaths occur. Although maternal and child mortality have dropped nearly 50 % since the 1990s [2], progress is varied between regions and neighbouring countries. For example, Peru met both MDG4 and MDG5, whereas Malawi met MDG4 but not MDG5 [3]. Accordingly, Countdown has supported a set of country case studies to improve understanding of the causes and processes that underpin or detract from achievement of MDG4 and MDG5. The aim of the case studies is to better understand the complex factors contributing to or detracting from progress in reproductive, maternal, newborn, and child health (RMNCH) in each of the selected countries over a period of about a decade, although the time frame of investigation varies by country [4]. The evaluation framework used to guide the Countdown case studies is presented in Fig. 1.

The past decade has seen an encouraging increase in the availability of data and development of methods for evaluating influence coverage levels including patterns of equity, and contextual variables impact on RMNCH. Measuring changes in policy and systems environments, including strength of policy to programme implementation, is also key to understanding which interventions have the greatest impact and to be able to anticipate future health gains [5–12]. Implementation data can also help in evaluating and improving progress toward specific RMNCH outcomes and intervention strategies [7, 10, 13–15]. However, a “black box” remains around how to assess the health systems inputs section of the evaluation framework in a systematic and standardised manner (Fig. 1), as the relationship between policy formulation and implementation, and changes in health systems inputs are unclear with limited scientific evidence. Though advances have been made on defining the stages of policy formulation [12], there is limited multi-country assessment tracking the policy formulation to implementation pathway, especially from lower and middle-income countries, impeded by a lack of HSP definitions, data and standardised tools. To date, more quantitative approaches have been used to assess HSP changes [16, 17], than qualitative approaches [18]. A recent systematic review on implementation strength concluded that currently there is no consensus on measuring implementation strength of RMNCH interventions with consistent definitions and methodologies [19].

With increasing availability of HSP data, especially at national level, from health management information systems (HMIS) and other routine data collections systems being put into place, there is an opportunity to routinely track RMNCH policy formulation to implementation including health systems strengthening activities within and across countries and to develop methodologies to link HSP to RMNCH outcomes on a regular basis. This paper’s objectives are to describe a standardised set of tools developed by Countdown to systematically analyse national policy formulation for RMNCH and progress to implementation, assessing similarities and differences between countries and programmes across the continuum of care. These tools assess whether HSP change is happening, supporting analyses which can then be used to ask whether and why or why not it is occurring. Results from two Countdown country case studies, Tanzania and Peru, are described to illustrate outputs from the HSP tools, as these two country teams were integral in piloting and refining the tools. The HSP tools include:

1. *Policy and Programme Timeline Tool* to identify what policies and major systems changes were introduced for RMNCH over time or the lack thereof from 1990 to present;
2. *Health Policy Tracer Indicators Dashboard* to track a set of key tracer RMNCH policy indicators as tracked on Countdown country profiles;
3. *Health Systems Tracer Indicators Dashboard* to assess key tracer health systems indicators as per Countdown country profiles; and
4. Programme implementation assessment using geographical mapping with implementation readiness barometers to assess health system readiness to implement RMNCH interventions.

## Methods

**Fig. 2:**
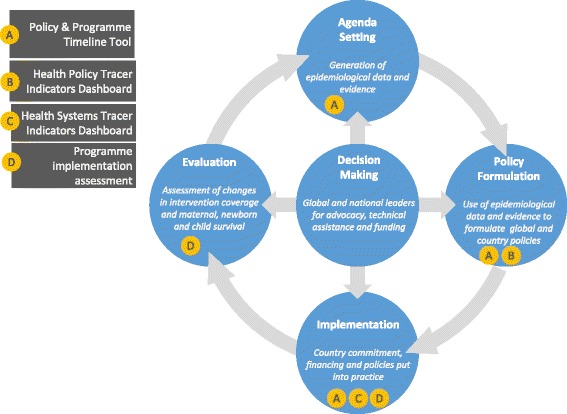
Countdown to 2015 health systems and policies tools (*A* to *D*) and where they link to the policy heuristic

Countdown used the four phases of the ‘stages heuristic’ of the public policy process [20] to guide the development of the HSP tools and approaches. Figure 2 shows the HSP tools and approaches in relation to the four phases of the policy heuristic. *Agenda setting*, the first phase, is the stage where a handful of the hundreds of problems that exist gain the attention of social actors and national decision-makers. *Policy formulation*, the second phase, refers to deliberation surrounding policy alternatives, and the enactment of authoritative decisions concerning which of these to adopt. *Policy implementation*, the third phase, involves the execution of policy. Finally, the *evaluation* phase assesses policy impact. These tools do not assess the cross-cutting dimension of policy change related to the *decision-making* phase of the heuristic, i.e. governance, power and partnerships, key to assessing the “how” and “why” of policy change.

Countdown developed HSP tools to standardise the type of data collected (inputs), methods for assessing (process) and presenting these data (outputs) for each of the countries included in the portfolio of the country case studies, to assess and describe HSP factors that have contributed to change, or lack thereof, in RMNCH in a country, and to enable cross-country comparison of policy and systems changes.

## Results

### Policy and programme timeline tool

#### Aim

The Policy and Programme Timeline Tool (Additional file 1) aims to assess the agenda setting component of the policy heuristic. This tool was developed to provide an overall view of health policies and programs of a country by examining changes in RMNCH policy, programs, and implementation from 1990 to the current year.

#### Data inputs

The tool is based on the Timeline tool developed for the *Health Policy and Planning* supplement on a multi-country evaluation of progress in newborn survival, policy and programmes [16]. The tool was adapted for use in Countdown case studies by broadening its scope to include macro health systems, health system building blocks and high impact policies and research specific to RMNCH.

#### Tool

**Fig. 3:**
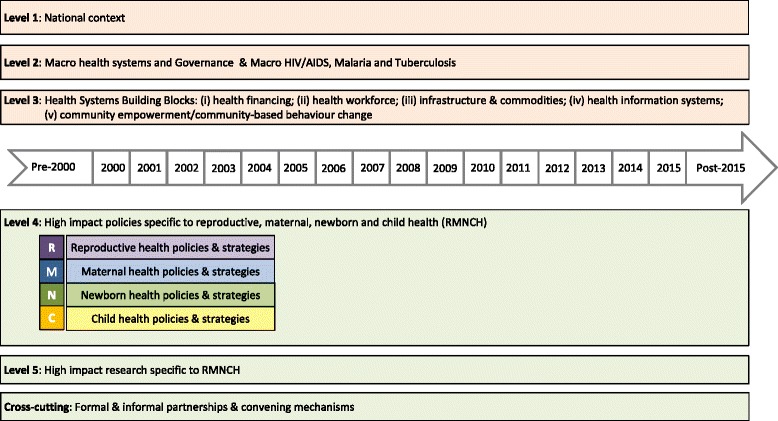
Components of the Countdown to 2015 policy and programme timeline tool

The Policy and Programme Timeline Tool (Additional file 1) spans across the following five levels: (i) national context; (ii) macro health systems and governance; (iii) health system building blocks; (iv) high impact policies specific to RMNCH; (v) high impact research specific to RMNCH; and a cross-cutting component focused on partnerships and convening mechanisms. Figure 3 provides a detailed overview of the sub-components of the Policy and Programme Timeline tool.

#### Process to apply the tool

Table 1 provides an overview of the steps required to complete and analyse the Policy and Programme Timeline Tool, with detailed methods specified in Additional file 2. Country teams draft the initial timeline, which is then reviewed by purposively selected stakeholders in-country in order to build consensus on the documentation of change in RMNCH. Once finalised, the timeline showcasing data across the tool’s five levels is synthesised and used for country-specific analyses as well as for comparison across countries as relevant.

**Table 1:** Standard process for completing the Countdown to 2015 policy and programme timeline

	Steps	Task(s)	Who
1	Drafting the policy and programme timeline	Country team to fill in the Policy and Programme Timeline Tool	Led by country teams with technical support as relevant
2	Country-specific analysis of policy and programme timeline	Country team to approach country partners/stakeholders to share the Policy and Programme Timeline and to use standardised questions as per the tool’s protocol to build consensus on what has changed and what has had the most impact on RMNCH via policy, programs, and implementation in their country	Led by country team, country partners/stakeholders and in some cases, additional support
3	Synthesising results from policy and programme timeline analysis	▪ Analysis of common themes across countries as relevant	Led by country teams with additional technical and graphic support as relevant
		▪ Draft standardised graphics representing analysis results for use in journal articles, policy briefs and/or other dissemination outputs as relevant	

#### Outputs

**Fig. 4:**
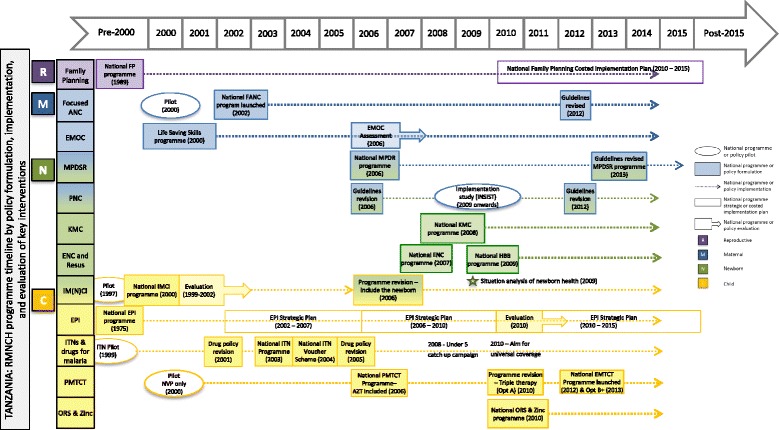
Countdown to 2015 policy and programme timeline tool: an example from Tanzania. *Source*: Adapted from Afnan-Holmes et al. 2015 [49]

**Fig. 5:**
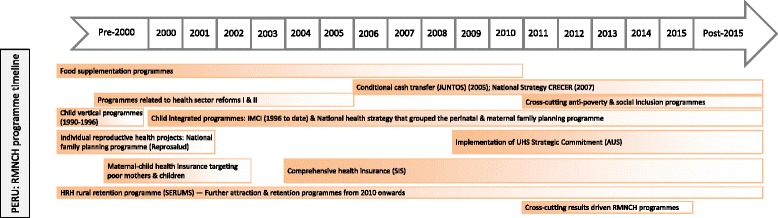
Countdown to 2015 policy and programme timeline tool: an example from Peru. *Source*: Adapted from Huicho et al. 2016 [60]

Results from Tanzania and Peru (1990–2014) are presented in Figs. 4 and 5, respectively. Tanzania’s RMNCH environment and policy formulation to programme implementation pathway is complex. Child health received consistent attention, focusing on increasing coverage of high-impact interventions at lower health system levels, with recent funding increases. Maternal health had high priority since mid-1990s, with variable implementation, targeting higher health system levels. Newborn health only received attention since 2006 yet is scaling up at facility and community levels. Reproductive health lost momentum from 2000–2005, with recent re-investment.

In contrast, Fig. 5 shows that Peru started with standalone RMNCH programmes, and has since moved to integrated policies and implementation, to combine multi-sectoral, anti-poverty and RMNCH programmes. The introduction of vertical childbirths and provision of waiting houses for pregnant women are an example of pro-poor and human rights-based approaches to close the health facility coverage gap for rural births.

### Health policy tracer indicators dashboard

#### Aim

The Health Policy Tracer Indicators Dashboard (Additional file 3) was developed to document, in a comparable way, 11 RMNCH tracer policy indicators reported in the Countdown country profiles to produce the Countdown Policy Dashboard for the country. This tool was developed to provide a systematic overview of the policy tracer indicators across the RMNCH continuum of care for a country by examining changes in these indicators from 1990 to present. The tool assesses and describes which RMNCH interventions have been translated into national-level policies (i.e. policy formulation component of the heuristic), and its visual output uses a “traffic light” colour coding system to illustrate if a policy has been fully (green) or partially (yellow) adopted, or does not exist (red).

#### Data inputs

The tracer indicators cover different areas of policies across RMNCH continuum of care. Data sources include the World Health Organisation (WHO) Maternal, Newborn, Child and Adolescent (MNCAH) Systems and Policy Survey, and specific sources e.g. the WHO abortion database and International Labour Organisation (ILO) maternity protection legislation database.

#### Tool

The Health Policy Tracer Indicators Dashboard (Additional file 3) assesses and describes the formulation of the following 11 policy tracer indicators, tracked by Countdown on country profiles, across the continuum of care from 1990 to present:

- Family planning for adolescents
- Legal status of abortion
- Midwives authorised for specific tasks
- Maternity protection (Convention 183)
- Maternal deaths notification
- Postnatal home visits in the first week after birth
- Kangaroo Mother Care for low birth weight newborns
- Antenatal corticosteroids as part of management of preterm labour
- International code of marketing of breastmilk substitutes
- Community treatment of pneumonia with antibiotics
- Low osmolality oral rehydration salts (ORS) and zinc for management of diarrhoea

The policy tracer indicators have been selected for global tracking and inclusion on the Countdown country profiles by the Countdown HSP Technical Working Group. These indicators are reviewed periodically and modified according to the latest evidence base. Definitions for the policy tracer indicators, including what constitutes a fully or partially adopted policy, are available in the Additional file 2.

#### Process to apply the tool

For each of these policy tracer indicators and specific components, respondents, e.g. relevant policy experts, stakeholders and implementers, are requested to review data sources and national policy documents and to select “Yes” or “No” from a dropdown menu if the policy or specific component exists. If the policy exists, respondents are requested to select “Yes” under the period when the policy was endorsed. These data are then presented in the Policy Dashboard, a visual representation of the data, which are reviewed by stakeholders in country in order to build consensus on the documentation of changes in selected RMNCH tracer policy and systems indicators. The tool's protocol with detailed methods is available in Additional file 2.

#### Outputs

**Fig. 6:**
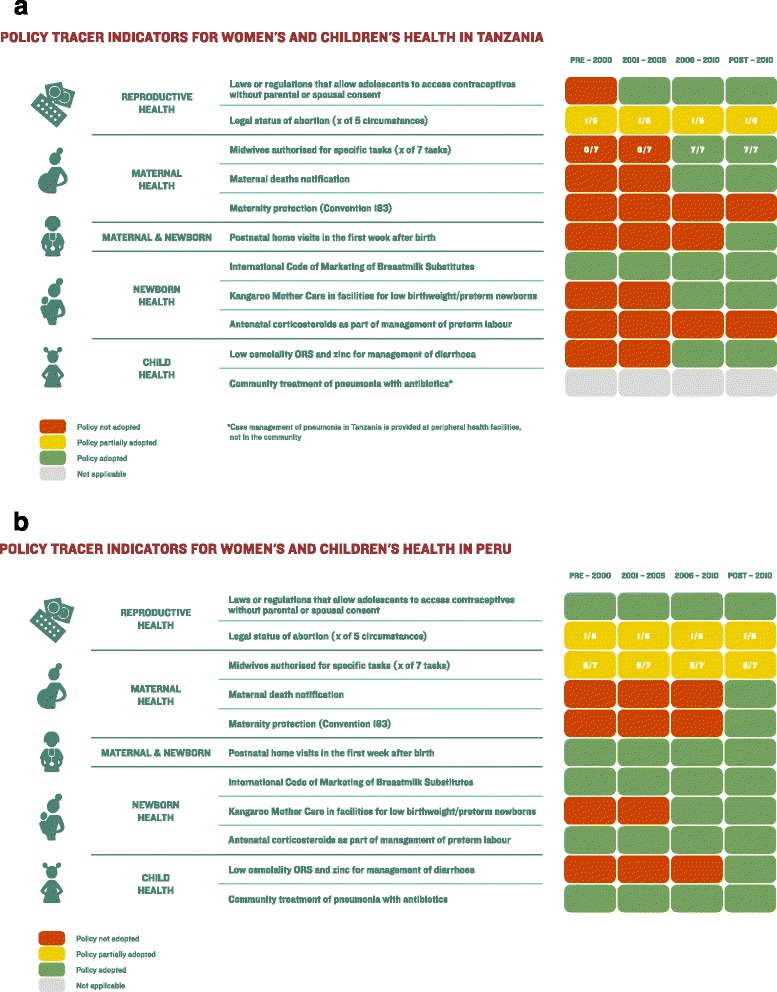
Countdown to 2015 health policy tracer indicators dashboard tool: **a**. An example from Tanzania. *Source*: Adapted from Afnan-Holmes et al. 2015 [49]; and **b**. An example from Peru. *Source*: Adapted from Huicho et al. 2016 [60]

The Health Policy Tracer Indicators Dashboard for Tanzania shows a mixed picture for RMNCH policy formulation since 2000 (Fig. 6a). Tanzania has adopted seven of the 11 policy tracer indicators since 2010, such as laws allowing adolescents to access contraceptives without parental or spousal consent and Kangaroo Mother Care in facilities for low birthweight and preterm newborns. However, gaps remain in policies related to circumstances under which abortion is allowed, task shifting for midwives, maternity protection in national law and practice (Maternity Protection Convention, 2000 [no. 183]) [21] and antenatal corticosteroids.

In contrast, the Health Policy Tracer Indicators Dashboard for Peru (Fig. 6b) shows that it has adopted nine of the 11 policy tracer indicators since 2010 including maternal death notification, antenatal corticosteroids and maternity protection. Peru has partially adopted the remaining two policy tracer indicators, with only one of five circumstances adopted for legal status of abortion, and midwives authorised for five of seven tasks.

### Health systems tracer indicators dashboard

#### Aim

The Health Systems Tracer Indicators Dashboard (Additional file 4) was developed to assess and describe, in a comparable way, selected RMNCH systems tracer indicators reported in the Countdown country profiles. This tool assesses and describes key components of the health system necessary to implement RMNCH policies, and focuses on assessing the policy implementation component of the heuristic. The tool was developed to provide a systematic overview of four systems tracer indicators of a country across the RMNCH continuum of care by examining changes in these indicators from 1990 to the current year. The tool’s visual output uses a “traffic light” colour coding system to illustrate if each systems indicators has been fully (green), partially (yellow), or not achieved (red).

#### Data inputs

These tracer indicators cover key dimensions of the health system essential for RMNCH including the WHO health system building blocks. Data sources include the WHO MNCAH Systems and Policy Survey for costed plans, a number of sources including the "USAID Deliver Project, World Health Organisation, International Consortium for Emergency Contraception" and the Chlorhexidine Working Group for essential commodities, emergency obstetric care (EmOC) surveys by WHO, United Nations Population Fund (UNFPA) and the Averting Maternal Death and Disability Programme (AMDD), and WHO Global Health Observatory for health workforce.

#### Tool

The Health Systems Tracer Indicators Dashboard (Additional file 4) tracks the implementation of the following four systems tracer indicators across the continuum of care from 1990 to present:

- National strategy/plans of action to improve RMNCH
- Integration of selected lifesaving commodities in essential medicines and supplies list
- Density of health professionals
- National availability of EmOC services

The systems tracer indicators have been selected for global tracking and inclusion on the Countdown country profiles by the Countdown HSP Technical Working Group, and are reviewed periodically and modified according to the latest evidence base. Definitions and data sources for these indicators are available in the Additional file 2.

#### Process to apply the tool

To use the Tool, respondents answer specific questions and collate the data within the tool. Data reported for “Density of health professionals” and “National availability of emergency obstetric care services” are then compared to globally agreed benchmarks. Lastly, all data within the tool are reviewed by stakeholders in country for validation purposes and to build consensus on their interpretation of changes in selected RMNCH tracer systems indicators. This tool's protocol with detailed methods is available in Additional file 2.

#### Outputs

**Fig. 7:**
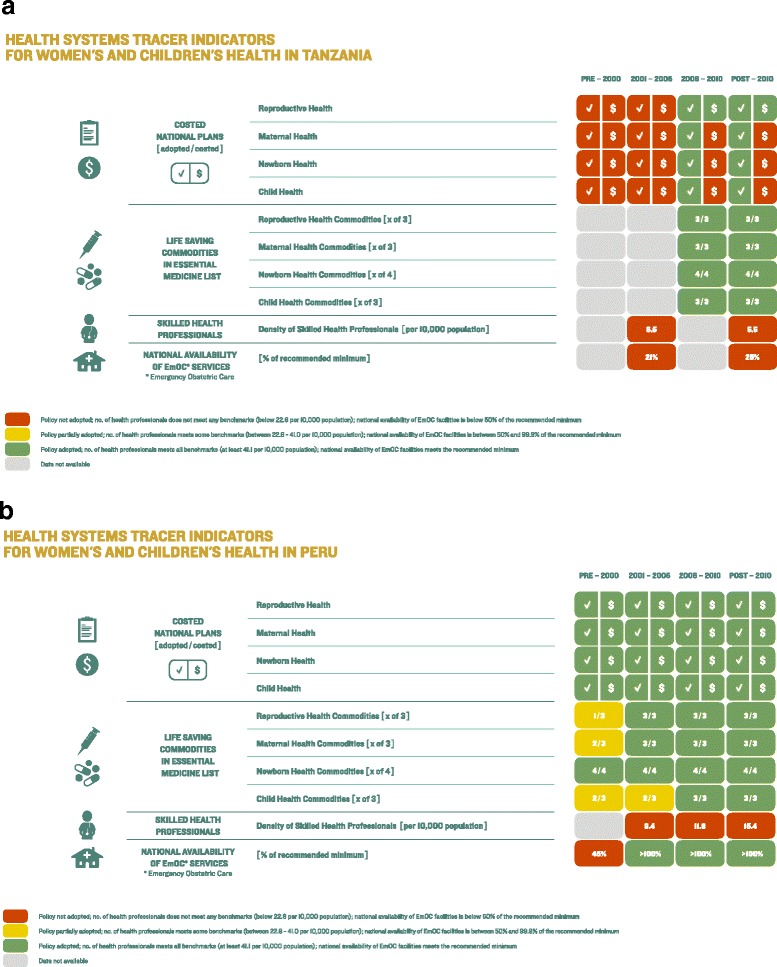
Countdown to 2015 health systems tracer indicators dashboard tool: **a**. An example from Tanzania. *Source*: Adapted from Afnan-Holmes et al. 2015 [49]; and **b**. An example from Peru. *Source*: Adapted from Huicho et al. 2016 [60]

The Health Systems Tracer Indicators Dashboard for Tanzania (Fig. 7a) and Peru (Fig. 7b) show contrasting pictures for the countries’ respective health systems. Peru has adopted and costed national RMNCH plans since before 2000, whereas Tanzania adopted national RMNCH plans in 2006 and has only costed the reproductive national plans to date, signalling financial gaps in implementation of MNCH national plans. Both Tanzania and Peru have included all lifesaving RMNCH commodities on their respective essential medicines and commodities list. In terms of health workforce, Peru has over twice as many skilled health professionals per 10,000 population as Tanzania (15.4 vs. 7.1, respectively), though both countries’ health workforce are far below the WHO minimum density threshold of 22.8 per 10,000 population [22]. At 45 % before 2000, Peru also met over twice the proportion of recommended minimum of national availability of EmOC services compared to 21 % in Tanzania during 2001–2005. However, recent data are available only for Peru, where EmOC assessments were performed since 2009 as part of wider obstetrical and newborn capabilities evaluations of health facilities, showing progressive improvement in availability of EmOC services [23, 24].

### Programme implementation assessment

#### Aim

Using the WHO health system building blocks as a foundation, we have developed a health system implementation readiness barometer, to be overlaid over a heat map of a country showing subnational variation in RMNCH outcomes. Its aim is to identify good and bad performing districts or regions, and to try to understand why there is subnational variation in implementation of RMNCH interventions, with a focus on service availability and service readiness.

#### Tool - Implementation readiness barometer

The implementation readiness barometer uses categorical data for four health systems building blocks — health financing, workforce, commodities, and facilities — pre-allocated to red, orange, yellow and green categories of strength based on international or national benchmarks as relevant. The circle shape of the barometer demonstrates the ‘wholeness’ of health system strength, i.e. all four health system components are interlinked to achieve readiness to implement a RMNCH intervention.

Barometers are overlaid on heat maps of health outcomes, which can be generated using software such as Arc GIS and Quantum GIS.

#### Process to apply the tool

Implementation readiness barometers can be constructed at the district, regional or zonal level of a country depending on availability of subnational data. As an example, we used Arc GIS 10.3 software to construct district-level maps in Tanzania with implementation readiness barometers to show variations in reproductive and child health outcome using DTP3 coverage and demand satisfied by modern methods of contraception as proxy indicators. The barometer was constructed using data from Tanzania’s Health Management Information System (HMIS) from Quarter 4 of 2014 [25, 26], the 2010 Tanzania Demographic and Health Survey (DHS) [27], the Human Resources for Health Country Profile (2012/13) [28], the 2012 Census [29], PMO-RALG Local Government Financial Report [30], and the Tanzanian Service Provision Assessment (SPA) Survey 2014 [31] to present regional-level variations for indicators based on the following four WHO health systems building blocks [32]: (i) *workforce*: skilled health workforce density per 10,000 population; (ii) *commodities*: availability of tracer drugs at health facilities; (iii) *financing*: per capita recurrent expenditure; and (iv) *infrastructure*: number of health facilities per 10,000 population.

**Fig. 8:**
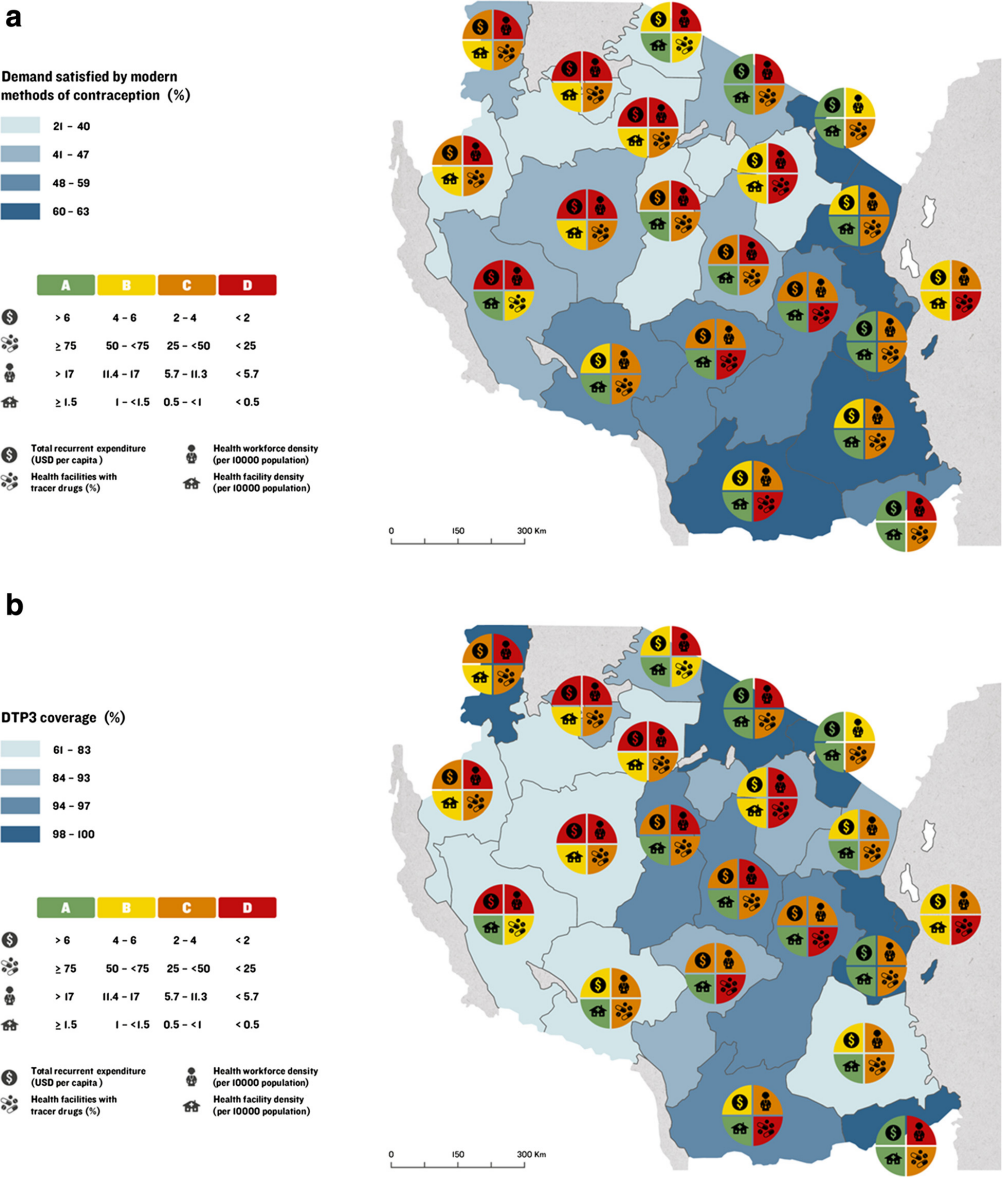
Programme implementation assessment: **a**. An example from Tanzania showing proportion of demand satisfied by modern methods of contraception vs. regional-level implementation readiness; and **b**. An example from Tanzania showing DTP3 coverage vs. regional-level implementation readiness

Data for the barometer were categorised based on data for each health systems indicator achieving a proportion of its respective benchmark, which were categorised as follows: (i) green: ≥ 75 %; (ii) yellow: 50 - <75 %; (iii) orange: 25 - <50 %; (iv) red: <25 %. For health workforce, the WHO minimum density threshold of 22.8 skilled health workers per 10,000 population was used as a benchmark (i.e. 100 %) in the construction of the barometer [22]. For health infrastructure, the global target of two public health facilities per 10,000 population recommended by WHO in its service availability and readiness assessment (SARA) guidelines [33] was used to construct the health infrastructure component of the barometer. No global benchmarks exist for total recurrent expenditure and commodities. Guidance for how much governments should spend at the regional or district levels does not exist, as available benchmarks on health expenditures from the Commission on Macroeconomics and Health and the WHO include central-level expenditures. In order to compare regions, we therefore grouped expenditures into four groups to show the diversity in funding levels and gave a green light to regions that have the highest expenditure levels. For commodities, we used ≥75 % as a benchmark for public health facilities with available tracer drugs. The legends in Fig. 8a and b provide an overview of how data for each indicator were categorised in the construction of the barometer for Tanzania.

#### Outputs

The barometers show mixed implementation readiness by region in Tanzania (Fig. 8a and b). None of the regions meet all of the benchmarks for the four health systems building blocks, and the north-western regions and Dar es Salaam region have the weakest implementation readiness compared to other regions. Across Tanzania, health workforce and availability of tracer drugs in health facilities do not exceed 50 % of the required threshold. Figures 8a and b show a contrasting picture of coverage of child and family planning interventions in Tanzania. DTP3 coverage in Tanzania is relatively high with a range of 61-100 % (Fig. 8b), whereas the proportion of demand satisfied by modern methods of contraception is lower with a range of 21-63 % (Fig. 8a).

## Discussion

This paper addresses the “black box” of HSP assessment by presenting a comprehensive set of standardised tools to use mixed methods to systematically analyse and describe progress of policy formulation to implementation to discuss how and why RMNCH changes took place within and across countries. Although these tools do not address the cross-cutting governance, power and partnerships dimension component of policy change; they focus on assessing if there are signs that RMNCH issues are being prioritised for consideration in policy agendas, being considered in policy formulation, and being moved towards implementation – to prompt discussion with a view to making further progress. This evidence is important in informing accelerated progress for ending preventable maternal, newborn and child mortality in the post-2015 era. The study represents a step forward in relation to previous efforts to document the role of health policy and systems on the evolution of RMNCH at country and sub-national levels, which used qualitative and quantitative approaches [34, 35], but did not include specific tools and minimum standards to track progress.

The Policy and Programme Timeline tool uses a chronological timeline to document and analyse what policies, programmes, and implementation strategies, and pivotal moments have changed for RMNCH in a country. This tool builds on the Policy and Programme Timeline tool developed by Saving Newborn Lives to capture changes in key policy, programme and research achievements influencing newborn survival at national level [16]. The policy and programme timeline tool developed by Countdown assesses variation for policies and strategies at the national level across the continuum of care, highlighting periods of policy intensity for RMNCH at the facility and community level. Outputs from this tool can be used to identify RMNCH policy gaps, target areas of policy to programme implementation for a more detailed assessment, and to compare policy changes with available data on trends in coverage, equity, and financing.

The Health Policy and Systems Tracer Indicators Dashboards are a standardised assessment of 11 RMNCH policy tracer indicators and four health systems tracer indicators reported on the Countdown country profiles and defined by the Countdown HSP Technical Working Group [36, 37]. These two dashboards chronologically assess the adoption of RMNCH policies and supporting health system building blocks at the national level in four time intervals from 1990 onward. More recently, a similar approach has been used by Saving Newborn Lives to develop a Benchmark Achievement Tool to assess readiness to scale up interventions for newborn survival [18], and by the Global Fund to assess progress toward programme objectives since 2004 [38]. The two Health Policy and Systems Tracer Indicators Dashboards assess progress in policy formulation and key components of the health system across the continuum of care rather than benchmarks for newborn survival, and are key to understanding how RMNCH policy and system environments have changed within and across countries.

The implementation readiness barometer is a novel way of visualising subnational variations in RMNCH outcomes with health service readiness and availability using the WHO health system building blocks. A recent review shows that geographical mapping is increasingly being used to visualise national and subnational data for women and children’s health [39], for example mapping health facilities with availability of RMNCH commodities using SARA data [40], mapping subnational variation in health workforce density, for example the density of Health Surveillance Assistants by population and access to health facilities in Malawi [41], and mapping big data for global health, for example by the Institute for Health Metrics and Evaluation for the Global Burden of Disease study [42]. The implementation readiness barometer presented in this paper builds on previous work by assessing more than one component of a health system against a RMNCH outcome. The construction of barometers using indicators for health financing, workforce, commodities, and infrastructure indicators paints a more comprehensive picture of health system strength, and can give clear indications of which components of the health system are succeeding or failing at the subnational level.

In addition to geographical mapping, bottleneck analysis using qualitative methods is another method that has been used to measure implementation strength. Dickson et al. built on analyses and evidence published previously in *The Lancet* Every Newborn Series [17], to adapt and apply the UNICEF Marginal Budgeting for Bottlenecks (MBB) tool in 12 Asian and African countries as part of the Every Newborn Action Plan process to synthesise bottlenecks hindering the scale up of maternal-newborn intervention packages across six health system building blocks [43]. This tool analysed bottleneck by health system building blocks for each maternal-newborn intervention with tracer indicators for each intervention, and used large focus group assessments to finalise results.

Implementation strength has also been measured previously using quantitative methods such as scores or rating systems based on mixed method data collection using interviews or focus groups. Bergh et al. developed and tested a monitoring model with quantitative indicators or progress markers to measure the progress of individual hospitals in the implementation of Kangaroo Mother Care in South Africa [44], and have since used the model for a multi-country assessment in four African countries [45]. This implementation assessment method showed variation in the quality of implementation of Kangaroo Mother Care between health facilities and across countries, and identified important factors for implementation of this intervention at the facility-level.

Benchmarks of health system readiness to implement integrated community case management of childhood illness (iCCM) have also been widely used [46]. These benchmarks have been complemented by indicators of iCCM implementation strength developed by a consortium under leadership of the Institute of International Programmes (IIP) and Johns Hopkins University [5]. The benchmarks and the indicators are based on the WHO health system building blocks and their application is widely promoted by WHO, UNICEF and partners in planning, management and review of maternal, newborn and child health programs. Nevertheless, collection of high-quality real time data remains a challenge.

Additional approaches to HSP analysis include Systems Thinking and Realist Review, which rely heavily on qualitative information [47, 48], take into account people, power and partnerships, and focus on the need to know at health system level not only what works but also for whom and under what circumstances, as well as to understand intended and unintended consequences resulting from the implementation of complex policy interventions. None of them include the use of specific tools, suggesting these HSP tools can be used to complement other analytic approaches.

### Strengths, limitations and future research

These are the first HSP tools developed for application across a range of lower and middle-income countries in Africa, Asia and Latin America, and can be used in other settings to analyse and describe HSP dynamics and changes in a country as related to implementation of RMNCH interventions, enabling cross-country comparisons. However, limited data availability both at national and subnational level remain a barrier to analysing HSP changes and implementation. These tools make progress in linking changes in impact indicators to changes in policies and programmes; however, there is a need to build on these tools to further develop mixed methods to identify key HSP factors and also attributable fractions of mortality decline for individual RMNCH policies and related programmes.

These HSP tools can also be used to prompt national policy dialogue. For example, the HSP tools were used as part of the Countdown Tanzania case study analysis, with results used to inform accelerated focused action towards the end of the MDGs and contributing to ending preventable maternal, newborn, and child deaths by the end of the Sustainable Development Goals in 2030 [49]. A policy brief with key messages on who is being left behind and where to focus efforts informed the evidence based Sharpened One Plan (2014 – 2015) [50]. The policy brief and the Plan were disseminated at a high level event attended by His Excellency President JM Kikwete of Tanzania.

The policy heuristic is the theoretical framework used to underpin these HSP tools. However, they are limited in assessing the implementation stage of the heuristic, signalling the need for the development of standardised metrics for strength of programme implementation. Additionally, these HSP tools do not capture information on power, partnerships, leadership and champions and the important role they play in influencing all stages of the policy heuristic, i.e. agenda setting, policy formulation, implementation and evaluation, to understand why and how policy change took place [51–54].

Furthermore, the policy heuristic framework has been critiqued by Sabatier et al. for presuming a linearity to the public policy process that does not exist in reality, for postulating neat demarcations between stages that are blurred in practice, and for offering no propositions on causality [55]. For example, the Countdown HSP tools document *when*, but not *why* changes in policies and programmes took place within and across countries, which raises the question of whether a linear approach to understanding policy adoption is appropriate, or if it should be combined with a political economy approach that recommends that policy adoption and decision making involves more than just evaluation of scientific evidence. Nevertheless, Walt et al. defend the heuristic as it offers a useful and simple way of thinking about the entire public policy process, and helps researchers situate their research within a wider framework [52].

When developing tools and metrics for HSP analysis, we also need to consider context-specific issues when setting-up reference standards, e.g. when considering facility-based versus community-based RMNCH interventions. For example, the latter may be privileged by some countries while the former may be considered a priority in health systems like in Peru.

There is a need for the development and validation of globally-agreed standards for WHO health systems building blocks to inform evidence-based benchmarking. We constructed benchmarks for health financing and commodities indicators due to lack of international standards. Varying benchmarks exist for health workforce and facilities, with little agreement on global standards, e.g. WHO’s minimum density threshold is 23 skilled health professionals per 10,000 population(34), whereas the International Labour Organisation recommendations vary from 35 [56] to 41 health workers per 10,000 population [57]. Grading data into traffic light categories can be reductive; for example, even the “green lighted” regions do not necessarily have the adequate amount of human resources, financing, RMNCH commodities or health facilities. However, the traffic light system of health system readiness is useful for national and subnational planning and assessment, and complements the new RMNCH scorecards in Tanzania and globally [58, 59].

None of the approaches used to date to assess RMNCH intervention implementation have been assessed comparatively or synthesised to perform an in-depth assessment of policy formulation to programme implementation pathways. However, with national and subnational-level data becoming increasingly available via establishment of routine data collections systems, there is a need to develop analysis methods to address this “black box” of HSP science. At the subnational level, data analysis methods need to be developed and applied to identify “stronger” and “weaker” performing districts on RMNCH outcomes and health systems indicators, and to evaluate subnational variation in implementation of the RMNCH interventions, with a focus on service availability and readiness. These novel subnational data analysis methods should link implementation strength to health outcomes, and be complemented with analyses of governance, power and partnerships to fully understand how and why policy change took place; thus allowing for assessment of national policy formulation to implementation within and across countries.

## Conclusions

The Countdown HSP tools are the first mixed method assessment to analyse when changes in policies and programmes took place within and across countries. Further work is needed in developing standardised approaches to measure the implementation strength of programmes, which is critical to attribute health outcomes to interventions, and to anticipate outcomes of future interventions. This evidence base is key to understanding how countries are able to make progress in ending preventable maternal, newborn and child deaths, and can provide important lessons to guide countries in their efforts to reach the Sustainable Development Goals (Table 2).

**Table 2:** Key messages

***Key messages***
1. **“Black box” of health system and policy (HSP) assessment:** This paper presents a standard set of tools to systematically describe policy formulation for reproductive, maternal, newborn and child health and assess changes in programmes and implementation within and across countries, and over time. We adapted the tools from the ‘stages heuristic’ of the policy process (i.e. agenda setting, policy formulation, policy implementation and evaluation).
2. **National and subnational change:** To date, these tools have been mainly applied at national level to assess HSP change over time and by programme, but HSP assessment would be even more valuable and needs further development at subnational level, particularly to better understand variation in health outcomes.
3. **Implementation strength:** Metrics to track strength of implementation need more work to develop, particularly to be comparable between programmes and across geographies. These metrics would be especially valuable if linked to GIS data and considering finance, human resources and domains for service readiness.
4. **Research gaps:** Presentation of data using consistent visualisations may help with interpretation of complex policy changes, but further assessment of perceptions and use of such visualisations would be valuable. Future research should also include analyses of power, partnerships and governance to complement the HSP tools’ outputs by providing a deeper understanding of how and why policy change took place.

## Abbreviations

AMDD, Averting death and disability programme; EmOC, emergency obstetric care; HMIS, health management information system; HSP, health systems and policy; ILO, International Labour Organisation; MBB, marginal budgeting for bottlenecks; ORS, oral rehydration salts; RMNCH, reproductive, maternal, newborn and child health; SARA, service availability and readiness assessment; SPA, service provision assessment; UNCoLSC, United Nations commission for life saving commodities for women and children; UNFPA, United Nations population fund; UNICEF, United Nations children’s fund; WHO, World Health Organisation

## Additional files

Additional file 1: Countdown to 2015 health systems and policy tools and protocols. (DOCX 241 kb) Additional file 2: Policy and Programme Timeline Tool. (XLS 39 kb) Additional file 3: Health Policy Tracer Indicators Dashboard. (XLS 23 kb) Additional file 4: Health Systems Tracer Indicators Dashboard. (XLS 18 kb)
