# Supplementary material for: Countdown to 2015 country case studies: systematic tools to address the “black box” of health systems and policy assessment
Source: BMC Public Health. 2016 Sep 12;16(Suppl 2):790. doi: 10.1186/s12889-016-3402-5 (PMC5025822; doi:10.1186/s12889-016-3402-5)
Supplement: Additional file 1: — Countdown to 2015 health systems and policy tools and protocols. (DOCX 241 kb) [file 12889_2016_3402_MOESM1_ESM.docx]

**Countdown to 2015 country case studies: systematic tools to address the “black box” of health systems and policy assessment**

***Additional file 1***

Table of Contents

[A. Countdown to 2015 Policy & Programme Timeline Tool 2](#_Toc439764836)

[A.1 Tool 2](#_Toc439764837)

[A.2 Protocol 2](#_Toc439764838)

[B. Countdown to 2015 Health Policy Tracer Indicators Dashboard 12](#_Toc439764839)

[B.1 Tool 12](#_Toc439764840)

[B.2 Protocol 12](#_Toc439764841)

[C. Countdown to 2015 Health Systems Tracer Indicators Dashboard 14](#_Toc439764842)

[C.1 Tool 14](#_Toc439764843)

[C.2 Protocol 14](#_Toc439764844)

[D. Countdown to 2015 health system and policy tracer indicators: Definitions and data sources 16](#_Toc439764845)

# Countdown to 2015 Policy & Programme Timeline Tool

## A.1 Tool

See Additional file 2.

## A.2 Protocol

**Countdown to 2015:**

**Policy and ProgramME Timeline Tool protocol**

“Big Picture” of National Policy Change for

Reproductive, Maternal, Newborn and Child Health

“Telling the Story”

______________________________________________________________________________

**Aim of the Countdown to 2015 (CD) Health System and Policy (HSP) Analysis**

The CD Policy and Programme Timeline Tool will be used will be used in conjunction with the health policy and systems tracer indicator dashboard tools as part of the qualitative analysis for CD Country Case Studies. The Countdown Policy and Program Timeline Tool will provide an overall view of health policies and programs of a country by examining changes in Reproductive, Maternal, Newborn and Child Health (RMNCH) policy, programmes, and implementation from 1990 to the current year. Country teams conducting CD Country Case Studies will draft the initial timeline, which will then be reviewed by stakeholders in country in order to build consensus on the documentation of change in RMNCH. Once finalised, the timeline will be synthesised and used for country-specific analyses as well as for comparison across the CD Country Case Studies.

The general aim of the Policy & Programme Timeline Tool is to understand through a standardised process with standardised content, what HSP factors have contributed to change (or the lack thereof) in RMNCH in each of the countries conducting a CD Country Case Studies.

CD aims to use the policy heuristics of (i) agenda setting; (ii) policy formulation; (iii) policy implementation; and (iv) policy evaluation as guiding principles in the development and analysis of all health policy and systems tools. The Policy & Programme Timeline Tool focuses on the agenda setting component of the heuristic.

**Objectives: CD Policy and Program Timeline Tool**

1. To use a chronological timeline to build consensus with country RMNCH partners (e.g. government, NGOs, UN agencies), and document and analyse what policies, programmes, and implementation strategies, pivotal moments, windows of opportunity have changed for RMNCH from 1990 to the current year.
2. To assess which inputs may have contributed to change in coverage and impact indicators from 1990 to the current year, and to identify important themes and stories of change and future gaps.
3. To assess the most important policy and program inputs for RMNCH and better understand how change occurred. If no change has occurred in coverage and impact indicators, the objective is to identify possible barriers or reasons for no change.
4. To draw lessons learned about policy change and scale up of RMNCH interventions that could be applied to accelerate progress for other public health interventions, or other countries and disseminate in a peer-reviewed paper

This protocol describes the:

1. Standard Tool; a Microsoft Excel spreadsheet for collating data; and
2. Standard Process; methods for conducting a country-specific analysis to complete the Policy & Programme Timeline Tool

**A. Standard Tool: CD HSP Policy and Program Timeline**

1. Columns
2. Rows (Level 1; Level 2; Level 3; Level 4; Level 5; All levels)
3. References and Acronyms
4. Descriptive data: Population and Gross National Income (GNI)
5. **Columns:**

The standard template includes four columns:

1. Pre - 2000
2. 2000 - 2005
3. 2006 - 2010
4. Post - 2010
5. **Rows**

The Policy and Program Timeline will include data on the following levels:

| **Level** | **Component** | **Sub-components** |
| --- | --- | --- |
| 1 | National Context | N/A |
| 2 | Macro Health Systems and Governance & Macro HIV/AIDS, Malaria & TB | Health Systems and Governance |
|  |  | HIV/AIDS, Malaria & TB |
| 3 | Health System Building Blocks | Health System Financing |
|  |  | Health Workforce |
|  |  | Infrastructure and Commodities |
|  |  | Participatory community empowerment /  Community-based behaviour change |
|  |  | Health Information Systems |
| 4 | High Impact Policies Specific to RMNCH | Reproductive health policies & strategies |
|  |  | Maternal health policies & strategies |
|  |  | Newborn health policies & strategies |
|  |  | Child health policies & strategies |
| 5 | High Impact Research Specific to RMNCH | N/A |
| All | Partnerships and Convening Mechanisms | Formal |
|  |  | Informal |

**NOTE**: Annex 1 includes a list of information required in each level of the Policy & Programme Timeline tool.

1. **References and Acronyms**

References

All policy documents, implementation strategies, situation analyses, research studies, etc. need to be cited at the bottom of the timeline. Include author name(s) (Last name, first name), title of document, place of publication, date of publication.

Acronyms

An acronym legend should be included at the bottom of the table or if preferred in a separate tab.

1. **Descriptive data: Population and Gross National Income (GNI)**

The first row of the table should include population and GNI data for one year in the 1990s and 2 spanning the 2000s. Specific data required for each of the three years are as follows:

- Total population (*n* million)
- Urban population (%)
- GNI per capita (USD $)

Please see section A.1 for the Policy & Programme Timeline Tool.

**B. Standard Process for completing the Policy and Programme Timeline**

The following table describes the 3 steps to complete a Policy & Programme Timeline:

|  | **Steps** | **Task(s)** | **Who** |
| --- | --- | --- | --- |
| 1 | Drafting the Policy and Programme Timeline | Country team to fill in the Policy and Programme Timeline Tool   - See Annex 1 for information required to fill the tool | Country teams with technical support as relevant |
| 2 | Country-specific analysis of Policy and Programme Timeline | Country team to approach country partners/ stakeholders to share the Policy and Programme Timeline and to use standardised questions as per the tool’s protocol to build consensus on what has changed and what has had the most impact on RMNCH via policy, programmes, and implementation in their country.   - See Annex 2 for guidance on conducting the analysis | Country team, country partners/ stakeholders and in some cases, additional support |
| 3 | Synthesising results from Policy and Programme Timeline analysis | - Analysis of common themes across countries as relevant - Draft standardised graphics representing analysis results for use in journal articles, policy briefs and/or other dissemination outputs as relevant - See Annex 3 for guidance on synthesising results, and Annexes 4 and 5 for examples of graphics | Country teams with additional technical and graphic support as relevant |

**Annex 1: Suggested content for completing CD Policy and Programme Timeline Tool**

- **NOTE:** It is likely that items will qualify for multiple rows, in which case include it in the first relevant row as your read from top to bottom. In addition, either include sufficient information so that it is clear that it could have been in two rows, or repeat in the second row.

**LEVEL 1: National context**

- This row will include information on non-health variables that may have affected RMNCH. For example, change in the political regime, humanitarian disasters, change in women’s status (look at MDG3 indicators over time); major economic changes and rapid urbanization.

**LEVEL 2: Macro Health Policies and Strategies**

***Health Systems and Governance***

- This row will include national level macro health policies, strategies, and plans which incorporate RMNCH e.g. National Health Plan, Health Sector Strategic Plan etc. Specifically, include relevant information on the following key components:
  - Right to the highest attainable standard of health
  - Universal access to healthcare and services
  - Integration of RMNCH into national health strategy and plan
  - National RMNCH strategies and implementation plans
  - RMNCH institutional arrangements
  - RMNCH programming includes a human rights based approach
  - Standards on quality of RMNCH care
  - Standards for RMNCH referral care
- More general national milestones such as committing to the Millennium Development Goals and national funded programs could also be included here if it is of national significance.

***HIV/AIDS, Malaria & TB***

- This row will include national level macro health policies, strategies, and plans relating to HIV/AIDS, Malaria & TB

**LEVEL 3: Health System Building Blocks**

- These rows will include important policies and programs that effect RMNCH according to four specific categories based on the WHO Health System Building Blocks:
  - Health System Financing, including relevant information on the following key components:
    - Sustainable financing of RMNCH
    - RMNCH resource allocation and expenditure
    - Elimination of financial barriers
    - RMNCH resource reporting and tracking
  - Health Workforce, including relevant information on the following key components:
    - Deployment and retention
    - Accreditation and certification
    - Authorisation of service provision and task shifting
    - Unmet needs for midwives/doctors/health service providers
    - RMNCH training curricular
    - RMNCH continuous skills training
    - Supporting supervision for all RMNCH health workers in the delivery of quality RMNCH care
  - Infrastructure and Commodities, including relevant information on the following key components:
    - Essential infrastructure in health facilities
    - Essential medicine supply and equipment list
    - Medicine and commodity security
    - Stock-out policies
    - Water sanitation and hygiene
  - Participatory community empowerment/Community-based behaviour change, including relevant information on the following key components:
    - Key participatory community empowerment strategies/policies specific to RMNCH
    - Key community-based behaviour change strategies/policies specific to RMNCH
  - Health Information Systems, including relevant information on the following key components:
    - Birth registration
    - Death notification
    - Death reviews
    - Well-functioning health information system and surveillance system for RMNCH
    - Defining key RMNCH indicators
    - National and subnational RMNCH targets
    - Data review process

**LEVEL 4: High Impact Policies Specific to RMNCH**

- This row will include policies and programs specific to RMNCH independently, including:

4.1 Specific reproductive health policies, strategies and reports, including:

4.1.1 Access to sexual and reproductive health services e.g. Injectable contraception - e.g. lowest cadres authorized to administer

4.1.2 Legal basis for safe abortion, and policies/programs for post abortion care

4.1.3 Commodities e.g. contraception stock-out policies

4.2 Specific maternal health policies, strategies and reports, including:

4.2.1 Antenatal Care (ANC) including Malaria and Sexually Transmitted Infections (STIs) in pregnancy

4.2.2 Care at birth, skilled attendance, % CEmONC/ BEmoNC compliant health centres, quality of care

4.2.3 Postnatal Care (PNC)

4.2.4 Death Reviews

4.3 Specific newborn health policies, strategies and reports, including:

4.3.1 Essential newborn care, Neonatal resuscitation e.g. Helping Babies Breathe (HBB)

4.3.2 Management of preterm babies, including Kangaroo Mother Care (KMC)

4.3.3 PNC for mothers and newborns and Exclusive breastfeeding

4.3.4 Care for sick newborns e.g. through IMCI or facility care

4.4 Specific child health policies, strategies and reports, including:

4.4.1 IMCI including diarrhoea and pneumonia case management, e.g. oral rehydration and zinc

4.4.2 Child nutrition and breastfeeding

4.4.3 Malaria prevention including bed nets and ITPi

4.4.5. Immunisation

4.4.6 PMTCT

**LEVEL 5: High Impact Research Specific to RMNCH**

- These rows will include high impact research that has affected the landscape of RMNCH within the specific country. Only list research studies that had a major national impact. Research in this section should be more focused on studies and influential papers or series outside the country that had an influence inside.
- **NOTE:** This section should not include items such as HSSP MTR, since that is part of policy. Also, other program assessments and reports should be in their respective RMNCH rows, not in this research section.

**ALL LEVELS: Partnerships and Convening Mechanisms**

- These rows will include information on formal and/or informal partnerships and convening mechanisms within the specific country.

**Annex 2: Guidance on Conducting Country-specific Analysis of Policy and Programme Timeline**

- **SPECIFIC TASKS:**
  - Countdown (CD) Country Team to approach country partners/stakeholders and share the Policy and Program Timeline to build consensus on what has changed in policy, programs, and implementation in their country.
  - CD Country Teams and key partners/stakeholders to work through the questions below, document findings and note where consensus is strong or weak from their country Policy and Program Timeline Analysis.
  - Suggest to country teams to record minutes of meetings with partners going through the initial review and discussions of the questions below and then systematically summarise those notes identifying major themes.
  - Since questions are grouped in themes, it might be useful to start with a general discussion on reproductive, maternal, newborn and child health (RMNCH) within country to review the timeline before delving into specific questions (listed below).
  - If key events/sources of influence emerge from discussion are not reflected in the timeline, then they should be added to the timeline
  - This meeting can be used as the forum in which to propose a country paper
  - CD Country Team to share updated draft with the CD Health Systems and Policy Technical Working Group
  - CD Health Systems and Policy Technical Working Group to review and provide technical support
- **Note:** Those countries writing papers that include this analysis should summarize results into the detailed outline of the qualitative section of their paper or create a one page summary.
- **Questions to guide the country specific analysis**

These questions aim to guide the country-specific analysis of the Policy and Program Timeline tool for the country papers and report. Suggested questions for each row in the tool focus on the *Agenda Setting* and *Partnerships* components of the policy heuristic:

1. **Agenda Setting**

- What have been key policies/program milestones for RMNCH in the country?
- What windows of opportunity were used (and what was result)?
- Were opportunities missed?
- What were strategic steps taken to bring attention to RMNCH?
- Has reproductive health ascended as an issue in political and policy attention? And if so, how?
- Has maternal health ascended as an issue in political and policy attention? And if so, how?
- Has newborn health ascended as an issue in political and policy attention? And if so, how?
- Has child health ascended as an issue in political and policy attention? And if so, how?
- Has increased attention to one aspect of RMNCH affected attention to another aspect of health along the continuum of care?
- What did [Country X] plan in terms of addressing policy barriers and/or mobilizing resources for RMNCH and what actually happened (or did not)?
- How did the relevant ministry/ies build technical capacity in RMNCH in [Country X]
- Looking at different causes of maternal, newborn and child deaths, identify if one cause has had more attention than the others. If so, why?
- In reviewing completed timelines, are there obvious gaps in terms of the spectrum of interventions for RMNCH in [Country X]
- What did [Country X] plan in terms of taking advantage of opportunities for integration along the continuum of care and what was done to test feasibility of integrating (or did not do)?
- Is there a Behaviour change or communication strategy for RMNCH?

1. **Partnerships**

- Who has played critical role in RMNCH, e.g. Steering committee, government leader, NGO, donor or UN agency, professional associations, specific individual champions, etc.?
- Name any champions and indicate how they were involved?
- Is there a stakeholder that has been essential and without which achievements could not have been possible?
- Has there been significant opposition to any RMNCH policies? From whom? What influence did that opposition have?
- Were there any south-to-south visits from [Country X] to another? What influence did such visits have on RMNCH policies?
- Did [Country X] also host visitors from another country? If so, did that have an influence on the RMNCH policies?

**Annex 3: Guidance on Synthesising Results from Country Specific Analyses on Policy and Programme Timelines**

| **I. Analysis of common themes across countries conducting Countdown Country Case Studies** | | |
| --- | --- | --- |
|  | Theme | Questions to guide country analysis across countries |
| 1 | Lessons learnt about pathways to scale | - - Did major challenges in national context (first row of timeline figure) interfere or facilitate the process of scale up? If so how?   - Advocacy – what works best for getting reproductive, maternal, newborn and child health (RMNCH) on the national agenda?   - Program – overall which programs moved fast and which did not? E.g. Kangaroo Mother Care (KMC). Why? Why not? Did policy change link to program implementation? What helped this or hindered it? E.g. training strategies etc.   - Research - what new evidence or research for RMNCH led to action? |
| 2 | Lessons learnt about levers for scale | - For countries with government coordinating mechanisms (e.g. steering committee, taskforce, etc.), did policy change move after establishment of such a committee? How did these committees function (composition, coordination, sphere of influence, integrated vs. specific to newborn health). Were they consistent or variable? Why? - Did partnerships with professional associations contribute? How? - Did the media and use of wider civil society, parliamentarians contribute? How? |
| **II. Draft Countdown standardized graphics reflecting the Policy and Program Analysis to be used in a scientific journal and policy brief** | | |
| - Select and incorporate key data on RMNCH policies and programs into a graphic that is suitable for the purpose and audience.   - See Annex 4 and 5 for examples of graphics. Annex 4 depicts a policy timeline generated from the Niger Countdown Country Case Study and published in the Lancet (Amouzou et al, 2012). Annex 5 depicts a policy timeline generated from the Decade of Change for Newborn Survival Country analysis conducted in Bangladesh and published in a policy brief (Rubayet et al, 2012). | | |

**Annex 4: Policy & Programme Timeline presented in the Niger Countdown Case Study published in the Lancet (Amouzou et al, 2012)**


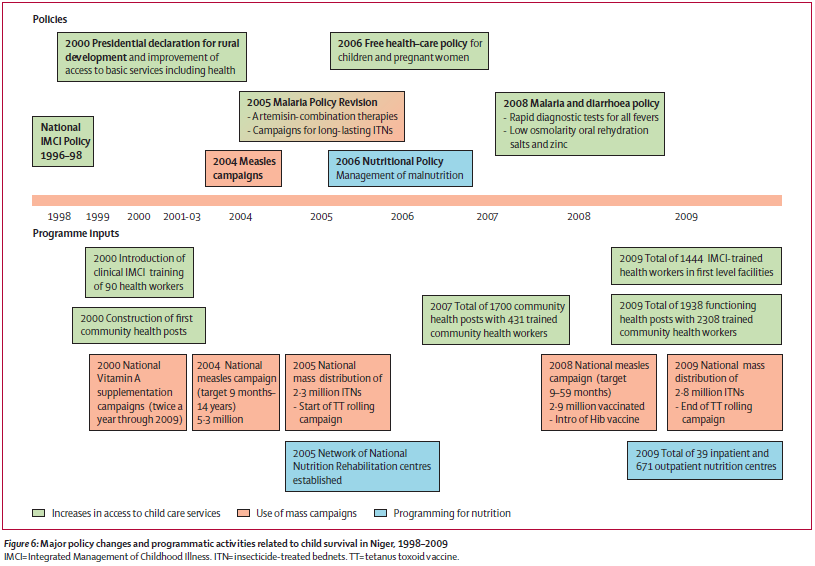


**Annex 5: Policy brief for A Decade of Change “Newborn Survival in Bangladesh”**


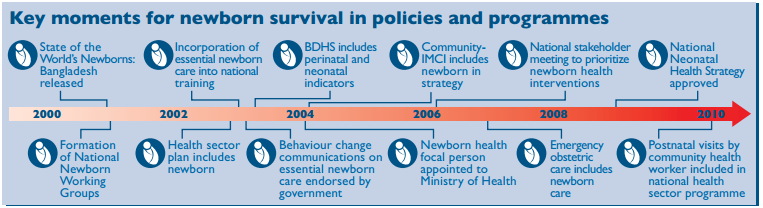


# B. Countdown to 2015 Health Policy Tracer Indicators Dashboard

## B.1 Tool

See Additional file 3.

## B.2 Protocol

**Countdown to 2015:**

**Health POLICY Tracer Indicators Dashboard protocol**

Policy Tracer Indicators from

the Countdown Country Profile

______________________________________________________________________________

**Aim of the Countdown Health Policy Analysis**

The Countdown (CD) Health Policy Tracer Indicators Dashboard will be used will be used in conjunction with the Policy and Programme Timeline tool and the Health Systems Tracer Indicators Dashboard as part of the qualitative analysis for CD Country Case Studies. The Health Policy Tracer Indicators Dashboard will document, in a comparable way, selected Reproductive, Maternal, Newborn and Child Health (RMNCH) tracer policies reported in the CD Country Profiles.

The general aim of the Health Policy Tracer Indicators Dashboard is to assess selected tracer RMNCH policy indicators to produce the CD Policy Dashboard for the country. This tool will provide a systematic overview of selected health policies tracer indicators of a country, by examining changes in these tracer indicators from 1990 to the current year. CD Country Case Study teams will answer specific questions and collate the data within the tool. These data will then be presented in the Policy Dashboard, a visual representation of the data, which will be reviewed by stakeholders in country in order to build consensus on the documentation of changes in selected RMNCH tracer policy and systems indicators.

Countdown aims to use the policy heuristics of (i) agenda setting; (ii) policy formulation; (iii) policy implementation; and (iv) policy evaluation as guiding principles in the development and analysis of all health policy and systems tools. The Health Policy Tracer Indicators Dashboard focuses on the policy formulation component of the heuristic.

**Objectives: CD Health Policy Tracer Indicators Dashboard**

1. To systematically assess the development and tracking over time of selected RMNCH tracer policy indicators and related health systems tracer indicators reported in CD Country Profiles
2. To visually represent policy tracer indicators in the form of the Policy Dashboard to bring about further interpretation and analysis

This protocol describes the standard tool – a Microsoft Excel spreadsheet for collating data – and the standard process, i.e. methods for conducting a country-specific analysis to complete the Health Policy Tracer Indicators Dashboard.

**Standard Tool: CD Health Policy Tracer Indicators Dashboard**

The Health Policy Tracer Indicators Dashboard investigates in a standardised manner the existence or not of selected RMNCH tracer policies at the national level monitored and reported by CD. See section B.1 for the tool in Microsoft Excel format.

The following eleven policies and their specific components are listed in the rows of the table:

1. Family Planning for adolescents
2. Legal status of abortion
3. Midwives authorised for specific tasks
4. Maternity protection (Convention 183)
5. Maternal deaths notification
6. Postnatal home visits in the first week after birth
7. Kangaroo Mother Care for low birth weight newborns
8. Antenatal corticosteroids for management of preterm labour
9. International code of marketing of breastmilk substitutes
10. Community treatment of pneumonia with antibiotics
11. Low osmolality ORS and zinc for management of diarrhoea

**Standard Process for completing the CD Health Policy Tracer Indicators Dashboard**

For each of these policies and specific components, respondents are requested to select “Yes” or “No”^^[[1]](#footnote-1)^^ from a dropdown menu if the policy or specific component exists. If the policy exists, respondents are requested to select “Yes” under the period when the policy was endorsed.

- **NOTE**: For some countries, there is no difference between *guidelines* and *policies*, so select “yes” from the dropdown menu for relevant cells if a related guideline is in place but not a policy.

Please ensure that each policy has a reference, ideally with a hyperlink, in the “**Reference**” column.

Please make notes related to the policies or its specific components sparingly under the “**Comments**” column whenever relevant.

# C. Countdown to 2015 Health Systems Tracer Indicators Dashboard

## C.1 Tool

See Additional file 4.

## C.2 Protocol

**Countdown to 2015:**

**Health Systems Tracer Indicators Dashboard protocol**

Policy Tracer Indicators from

the Countdown Country Profile

______________________________________________________________________________

**Aim of the Countdown Health Systems Analysis**

The Countdown (CD) Health Systems Tracer Indicators Dashboard will be used will be used in conjunction with the Policy and Programme Timeline tool and the Health Policy Tracer Indicators Dashboard as part of the qualitative analysis for CD Country Case Studies. The Health Systems Tracer Indicators Dashboard will document, in a comparable way, selected Reproductive, Maternal, Newborn and Child Health (RMNCH) tracer systems indicators reported in the CD Country Profiles.

The general aim of the Health Systems Tracer Indicators Dashboard is to assess selected tracer RMNCH systems indicators to produce the CD Systems Dashboard for the country. This tool will provide a systematic overview of selected health systems tracer indicators of a country, by examining changes in these tracer indicators from 1990 to the current year. CD Country Case Study teams will answer specific questions and collate the data within the tool. These data will then be presented in the Systems Dashboard, a visual representation of the data, which will be reviewed by stakeholders in country in order to build consensus on the documentation of changes in selected RMNCH tracer systems indicators.

Countdown aims to use the policy heuristics of (i) agenda setting; (ii) policy formulation; (iii) policy implementation; and (iv) policy evaluation as guiding principles in the development and analysis of all health policy and systems tools. The Health Systems Tracer Indicators Dashboard focuses on the policy formulation component of the heuristic.

**Objectives: CD Health Systems Tracer Indicators Dashboard**

1. To systematically assess the development and tracking over time of selected RMNCH systems tracer indicators reported in CD Country Profiles
2. To visually represent system tracer indicators in the form of the Systems Dashboard to bring about further interpretation and analysis

This protocol describes the standard tool – a Microsoft Excel spreadsheet for collating data – and the standard process, i.e. methods for conducting a country-specific analysis to complete the Health Systems Tracer Indicators Dashboard.

**Standard Tool & Process for Completion: CD Health Systems Tracer Indicators Dashboard**

The Health Systems Tracer Indicators Dashboard contains the following three tables:

**Table 1** tracks in a standardised way the existence or not of national strategy/ plans of action to improve RMNCH. Respondents will select “Yes” or “No” from a dropdown menu if the strategy and/or plan exists and is costed. If the strategy/plan exists, then enter the years the strategy document covers under the columns labelled “Start Year of the Strategy” and “End year of the Strategy.”

- Note: Often the strategies and plans for RMNCH are not a standalone document, but rather integrated with each other in different combinations. Answer “Yes” if that is the case and in the column “Comments” mark the name of strategy and provide full reference in the “Reference” column.

Please ensure that each policy/strategy has a reference, ideally with a hyperlink, in the “**Reference**” column.

Please make notes related to the policies/strategies or its specific components sparingly under the “**Comments**” column whenever relevant.

**Table 2** tracks integration of selected Lifesaving commodities in Essential medicines and supplies list (EML).

Respondents are requested to indicate by “Yes” or “No” if the specific medicine/supply is included in the EML. If the answer is “Yes”, please mark this under the period column when this medicine/supply was introduced in EML.

Please ensure that each policy/strategy has a reference, ideally with a hyperlink, in the “**Reference**” column.

Please make notes related to the policies/strategies or its specific components sparingly under the “**Comments**” column whenever relevant.

**Table 3** tracks progressive changes in the following two systems’ tracer indicators:

- Density of health professionals
- National availability of emergency obstetric care services

For each of these systems’ indicators, note the data, the year the data was reported and source of data.

If possible and available, please report more than one data point.

- Note: Table 3 provides columns for data collected at 3 different points. However, add more columns as needed if countries have data from additional years.

See Section C.1 for the tool in Microsoft Excel format.

# D. Countdown to 2015 health system and policy tracer indicators: Definitions and data sources

| ***Countdown to 2015 Health Policy Indicators*** | | | | | | | | | |
| --- | --- | --- | --- | --- | --- | --- | --- | --- | --- |
| **INDICATOR** | | **DEFINITION** | | | **CRITERIA FOR RANKING** | | | **DATA SOURCE** | **GLOBAL DATABASE** |
| Family planning for adolescents | | Laws or regulations allow adolescents (married or unmarried) to access contraception without parental or spousal consent. | | | Yes = legislation is available that allows adolescents to access contraception without parental or spousal consent. Partial = legislation is available that allows either married adolescents to access contraception without spousal consent or allows unmarried adolescents to access contraception without parental consent. No = no legislation is available that allows adolescents to access contraception without parental or spousal consent. | | | World Health Organization | Global Maternal Newborn Child and Adolescent Health Policy Indicator Survey by the World Health Organization Department of Maternal Child Adolescent Health |
| Legal status of abortion | | Legal grounds under which abortion is allowed. | | | Abortion allowed on the following grounds: 1 = to save a woman's life. 2 = to preserve physical health and above. 3 = to preserve mental health and above. 4 = for economic and social reason and the above. 5 = on request and above. R = in case of rape or incest. F= in case of foetal impairment. - = data are not available. | | | United Nations Population Division policy database | United Nations Population Division policy database http://esa.un.org/poppolicy/about_database.aspx (Accessed January 2014 |
| Midwives authorized for specific tasks | | Midwifery personnel are authorized to deliver basic emergency obstetric and newborn care. | | | Number of the seven lifesaving interventions tasks authorized: parental antibiotics, parenteral oxytocin, parental anticonvulsants, manual removal of placenta, removal of retained products of conception, assisted vaginal delivery, newborn resuscitation. | | | World Health Organization | Global Maternal Newborn Child and Adolescent Health Policy Indicator Survey by the World Health Organization Department of Maternal Child Adolescent Health |
| Maternity protection (Convention 183) | | Country has ratified International Labour Organization Convention 183 or has passed national legislation that is in compliance with the three key provisions of the convention (14 weeks of maternity leave, paid at 66% of previous earnings by social security or general revenue) | | | Yes = International Labour Organization Convention 183 ratified (maternity leave of at least 14 weeks with cash benefits of previous earnings paid by social security or public funds). Partial = International Labour Organization Convention 183 not ratified but previous maternity convention ratified (maternity leave of at least 12 weeks with cash benefits of previous earnings paid by social security of public funds). No = no ratification of any maternal protection convention. | | | International Labour Organization | International Labour Organization, NORMLEX Information System on International Labour Standards, at: https://www.ilo.org/dyn/normlex/en (Accessed March 2014) |
| Maternal deaths notification | | National policy has been adopted requiring health professionals to notify any maternal death to a responsible national body. | | | Yes = national policy adopted and implemented. Partial = national policy adopted but no systematic implementation. No = no national policy adopted. | | | World Health Organization | Global Maternal Newborn Child and Adolescent Health Policy Indicator Survey by the World Health Organization Department of Maternal Child Adolescent Health |
| **INDICATOR** | | **DEFINITION** | | | **CRITERIA FOR RANKING** | | | **DATA SOURCE** | **GLOBAL DATABASE** |
| Postnatal home visits in the first week after birth | | National policy recommending home visits to mother and newborn in the first week after childbirth by a trained provider have been adopted and implemented. | | | Yes = national policy or guidelines recommending postnatal home visits adopted and implemented. No = no national policy or guidelines on postnatal home visits adopted. | | | World Health Organization | Global Maternal Newborn Child and Adolescent Health Policy Indicator Survey by the World Health Organization Department of Maternal Child Adolescent Health |
| Kangaroo mother care for low birthweight newborns | | National policy recommends kangaroo mother care for low birthweight newborns. | | | Yes = national policy recommends kangaroo mother care for low birthweight newborns. No = national policy does not recommend kangaroo mother care for low birthweight newborns. | | | World Health Organization | Global Maternal Newborn Child and Adolescent Health Policy Indicator Survey by the World Health Organization Department of Maternal Child Adolescent Health |
| Antenatal corticosteroids for preterm labour | | National policy recommends antenatal corticosteroids for preterm labour. | | | Yes = national policy recommends use of antenatal corticosteroids for preterm labour. No = national policy does not recommend use of antenatal corticosteroids for preterm labour. | | | World Health Organization | Global Maternal Newborn Child and Adolescent Health Policy Indicator Survey by the World Health Organization Department of Maternal Child Adolescent Health |
| International Code of Marketing of Breastmilk | | National policy has been adopted on all provisions stipulated in International Code of Marketing of Breastmilk Substitutes. | | | Yes = all provisions stipulated in International Code of Marketing of Breastmilk Substitutes adopted in legislation. Partial = voluntary agreements or some provisions stipulated in International Code of Marketing of Breastmilk Substitutes adopted in legislation. No = no legislation and no voluntary agreements adopted in relation to the International Code of Marketing of Breastmilk Substitutes. | | | World Health Organization | World Health Organization and United Nations Children's Fund special data compilation |
| Community treatment of pneumonia with antibiotics | | National policy or guidelines authorizing case management of pneumonia in the community by a trained provider has been adopted and implemented | | | Yes = national policy or guidelines adopted on the identification and treatment of pneumonia by trained providers in the community. No = no national policy or guidelines on the identification and treatment of pneumonia by trained providers. | | | World Health Organization | Global Maternal Newborn Child and Adolescent Health Policy Indicator Survey by the World Health Organization Department of Maternal Child Adolescent Health |
| Low-osmolarity oral rehydration salts and zinc for management of diarrhoea | | National policy on management of diarrhoea with low-osmolarity oral rehydration salts and zinc has been adopted and implemented. | | | Yes = national policy or guidelines adopted on use of low-osmolarity oral rehydration salts and zinc for management of diarrhoea. No = no national policy or guidelines adopted on the use of low-osmolarity oral rehydration salts and zinc for managements of diarrhoea. | | | World Health Organization | Global Maternal Newborn Child and Adolescent Health Policy Indicator Survey by the World Health Organization Department of Maternal Child Adolescent Health |
| ***Countdown to 2015 Health Systems Indicators*** | | | | | | | | |  |
| **INDICATOR** | | **DEFINITION** | **CRITERIA FOR RANKING** | | **DATA SOURCE** | **GLOBAL DATABASE** | | |  |
| Costed national implementation plan for maternal, newborn and child health | | National plan for scaling up maternal, newborn and child health interventions is available and costed. | Yes = costed plan or plans to scale up maternal, newborn and child health interventions available at the national level. Partial = costed plan available for either maternal and newborn health or child health. No = no costed implementation plan for maternal, newborn and child health available. | | World Health Organization | Global Maternal Newborn Child and Adolescent Health Policy Indicator Survey by the World Health Organization Department of Maternal Child Adolescent Health | | |  |
| Reproductive lifesaving commodities in essential medicine list | | Emergency contraceptives, implants and female condoms are in the essential medicine list. | Number of the three listed commodities that are included in the essential medicine list. (*) indicates that information is incomplete for the given country due to lack of data on the three commodities and, that EML includes as basic minimum the number of commodities indicated. | | USAID Deliver Project  World Health Organization   International Consortium for Emergency Contraception | Special data collection from: Emergency Contraception in National Essential Medicines Lists http://www.cecinfo.org/custom-content/uploads/2014/04/ICEC_EC-in-EMLs_Apr-2014.pdf  World Health Organization. “National Medicines List/Formulary/Standard Treatment Guidelines.” Web site: www.who.int/selection_medicines/country_lists/en/index.html (accessed January 2015) Emergency contraceptives and implants information: U.S. Agency for International Development Deliver Project, http://deliver.jsi.com/dhome/whatwedo/commsecurity/csmeasuring/csindicators/csindicatordashboards (Accessed March 2014). Female condoms information: World Health Organization EML database www.who.int/medicines/publications/essentialmedicines (Accessed 1 July 2015) | | |  |
| Maternal lifesaving commodities in essential medicine list | | Oxytocin, misoprostol and magnesium sulfate are in the essential medicine list. | Number of the three listed commodities that are included in the essential medicine list. | | World Health Organization | Global Maternal Newborn Child and Adolescent Health Policy Indicator Survey by the World Health Organization Department of Maternal Child Adolescent Health | | |  |
| **INDICATOR** | | **DEFINITION** | **CRITERIA FOR RANKING** | | **DATA SOURCE** | **GLOBAL DATABASE** | | |  |
| Newborn lifesaving commodities in essential medicine list | | Injectable antibiotics, antenatal corticosteroids, chlorhexidine and resuscitation equipment are in the essential medicine list. | Number of the four listed commodities that are included in the essential medicine list. | | World Health Organization and the Chlorhexidine Working Group | Special data collection from:  Global Maternal Newborn Child and Adolescent Health Policy Indicator Survey 2012 and 2014 by the World Health Organization Department of Maternal Child Adolescent Health  and  The Chlorhexidine Working Group | | |  |
| Child lifesaving commodities in essential medicine list | | Amoxicillin, oral rehydration salts and zinc are in the essential medicine list. | Number of the three listed commodities that are included in the essential medicine list. | | World Health Organization | Global Maternal Newborn Child and Adolescent Health Policy Indicator Survey by the World Health Organization Department of Maternal Child Adolescent Health | | |  |
| Density of health workers | | Proportion of physicians, nurses and midwives who are available per 10,000 population. | Percentage | | World Health Organization | Global Health Observatory | | |  |
| National availability of emergency obstetric care services | | At least five emergency obstetric care facilities per 500,000 people, including one comprehensive and four basic emergency obstetric care facilities. (The breakdown of comprehensive and basic by population and geographic area is available in country assessment reports but not included in the Countdown. | Availability is expressed as a percentage of the minimum acceptable number of emergency obstetric care facilities. The minimum acceptable number of emergency obstetric care facilities (comprehensive and basic) is calculated by dividing the number of functioning emergency obstetric care facilities by the recommended number and multiplying by 100. To qualify as a fully functioning basic or comprehensive emergency obstetric care facility, a facility must provide a standard set of signal functions. | | Averting Maternal Death and Disability, United Nations Children’s Fund, United Nations Population Fund | Averting Maternal Death and Disability, United Nations Children's Fund, United Nations Population Fund special data compilation | | |  |

1. Responses to “Community treatment of pneumonia with antibiotics,” policy has a “not applicable” option in addition to Yes and No options. [↑](#footnote-ref-1)
